# 6th European Student Council Symposium (ESCS): overcoming obstacles to enhance virtuality, connectivity, inclusivity and community engagement

**DOI:** 10.12688/f1000research.40666.1

**Published:** 2021-01-22

**Authors:** Gabriel J. Olguín-Orellana, Sofia Papadimitriou, Alberto Langtry Yáñez, Pradeep Eranti, Rosario del Carmen Flores-Vallejo, Ana I. Castillo-Orozco, Kalaumari Mayoral-Peña, R. Gonzalo Parra

**Affiliations:** 1 Center for Bioinformatics, Simulations and Modeling, Universidad de Talca., Talca, Chile; 2 Vrije Universiteit Brussel, Brussels, Belgium; 3 Spanish National Cancer Research Center (CNIO), Madrid, Spain; 4 Université de Paris, Paris, France; 5 Department of Biomedical engineering and Mechatronics, Universidad del Valle de México, Campus Toluca, Toluca, Mexico; 6 Department of Human Genetics, Faculty of Medicine, McGill University, Montreal, Canada; 7 School of Engineering and Sciences, Tecnologico de Monterrey, Queretaro, Mexico; 8 European Molecular Biology Laboratory, Genome Biology Unit, Heidelberg, Germany

**Keywords:** student council, iscb, conference, virtual

## Abstract

This editorial summarises the organisation, activities, and scientific content of the 6th European Student Council Symposium (ESCS) organised by the International Society for Computational Biology Student Council (ISCB-SC). The event was held on September 6, 2020, as a satellite event preceding the ISCB’s  19th European Conference in Computational Biology. Both events were first planned to be held in-person in Sitges, Spain, but moved virtually as a strategy to face the SARS-CoV2 sanitary crisis. This completely unforeseen situation has posed several challenges that have been successfully addressed thanks to the robust ISCB Student Council community structure and the strong commitment of the organisers. Despite all the obstacles and challenges, we have found that virtuality has several advantages that can continue to be kept to improve in-person meetings in the future and make conferences more inclusive allowing a larger audience to participate.

## Introduction

The International Society for Computational Biology Student Council (ISCB-SC) is a network composed of students and young researchers of all academic degrees, most of them working in the fields of bioinformatics, computational biology, and related fields. The ISCB-SC was launched in 2004 with the aim to educate and promote the consolidation of the next generation of computational biologists as a unified global community ( Corpas 2005). In 2006, the ISCB-SC founded the Regional Students Group (RSG) program to gather its members locally with the purpose of leading scientific outreach events and promoting networking opportunities, educational resources, and career advice; while supporting political processes affecting science and education ( Macintyre, Michaut, and Abeel 2013).

Along with the Student Council Symposium, Latin American Student Council Symposium ( Parra et al. 2014), and Student Council Symposium Africa ( Souilmi et al. 2015), the ISCB European Student Council Symposium ( Francescatto et al. 2015) is one of the main flag events of the ISCB-SC, organised by the RSGs, volunteer students and early-career researchers who join the organising committee in each edition ( Parisi et al. 2019). The ESCS is biennially organised with the aim to (1) gather students and young researchers together to present their work within an international format; (2) generate opportunities that can contribute to the development of their scientific career; and (3) encourage interactions among students coming from different research institutes, providing them with a platform to socialize with their peers and senior researchers ( Cuypers et al. 2016).

## Meeting Format

Unlike any other previous edition of the ESCS, the 6 ^th^ European Student Council Symposium ( ESCS2020) was held completely virtually. It was a half-day event on Sunday, September 6, following the traditional structure of ISCB-SC previous symposia, trying to keep their spirit and structure, regardless of the changes in the format ( Hassan et al. 2018). The event included two keynote speaker lectures, six student full talks, four student flash talks, a poster session and a virtual social event. This time, we also organised a round table about mental health in academia which is a topic that has been gradually gaining more and more importance in the last years among Ph.D. students and postdocs around the world ( Evans et al. 2018).

## Keynote Speakers

The day opened with the keynote presentation of Dr. Patrick Aloy (International Computer Room Experts Association and Institute for Research in Biomedicine, Spain). For twenty years, Dr. Aloy has worked developing and implementing new technologies and algorithms to bridge the gap between theoretical models and experiments in different disciplines. In his talk “Extending the small molecules principles to all levels of biology”, Dr. Aloy presented the *“Chemical Checker”*, a resource that provides processed, harmonized, and integrated bioactivity data on small molecules, possible to be applied to anticipate failures in clinical trials. Moreover, his laboratory demonstrated *Chemical Checker* signatures can be used to reverse and mimic biological signatures of disease models and genetic perturbations.

In the evening, the oral presentations session was ended by the second keynote, Dr. Sofia Forslund (Max Delbrück Center for Molecular Medicine, Germany), with her lecture entitled *“Confounder-aware systems in medical approaches”* in which she explained how her group applies high-dimensional multi-omics data, including metadata on disease development, nutrition, and lifestyle, to achieve a highly accurate, quantitative understanding of host-microbiome interactions, that can be translated into personalized therapies for the future. Her group worked to understand the effects of prolonged antibiotic exposure to both, population and individual levels, and the role of antibiotics from food production in resistance developed by bacteria in the human gut. These computational tools have also allowed them to link the microbiome to type 2 diabetes, insulin resistance, and hypertension, and to assess the role of diet and medication in these diseases, with the goal of developing personalized medicine and nutrition platforms for complex pathologies of the host.

## Student Presentations

ESCS2020 featured 344 attendees from 19 different countries. From all submissions, six abstracts were accepted for oral presentations, four as flash talks and 55 as for poster presentations. Talks were selected by four reviewers who evaluated the potential impact of the submitted work in the audience. Reviewers were instructed to consider authors' gender parity as an important selection criteria.

### Oral presentations (12-13 minutes)

The student talks were started by Nikolina Sostaric, who reported how molecular dynamics (MD) help us to understand the functional roles of post-translational modifications (PTMs) in yeast proteins interactions. In her work, A MD based framework was applied for predicting short and long-range effects of PTMs on stability of proteome-wide interactions. These PTM sites are widely conserved in orthologous proteins suggesting that these results might be mirrored to other organisms.

In the next presentation, Miguel Rodriguez Galindo talked about a higher *de novo* mutability in exons compared to introns. *De novo* mutation data through the whole genome sequencing of trios were used to study these mutation rate variations. The exonic observed mutations were significantly higher than the expected mutations. In order to explain these differences, an expanded sequence context model was employed.

Loic Lannelongue presented the Green Algorithms, an open source online calculator that allows users to estimate the environmental impact of their computation including the running time or type of computing cores, among other computational features. This knowledge allows researchers to discuss the importance of quantifying the environmental impact of their calculations and working towards sustainability.

Next presentation led us to address multiple sequence alignments, which are used to find point mutations or understanding evolutionary relationships between descendants from a common ancestor. Edgar Garriga Nogales shared the regressive alignment, a new way to perform multiple sequence alignments (MSA), which is based on the match of the most dissimilar sequences by labelling the intermediate nodes and then computing the sub-MSAs with any existing aligner. Regressive alignment allows MSA of 1.4 million sequences improving the accuracy compared to other conventional MSAs.

In the following talk, Asma Yamani described a sequence-based approach based on machine learning techniques to classify protein-protein interactions (PPI) in activation/inhibition relationships. The study of signalling pathways is particularly important to disentangle many cell-related biological phenomena. PPI has been highly important to understand the signalling mechanisms that occur in cells.

The last oral presentation was delivered by Ina Maria Deutschmann who presented different network-based methods to infer relationships between microbes by associations across time and space. Networks are composed of nodes (microbes) and edges (co-presence or exclusion relationships). In her work, Ina Maria showed how these methods were able to determine both, the edge prevalence, which was higher for deep ocean in comparison to ocean surface, and the alignment of the network similarity with environmental factors.

### Flash talks presentations (2-3 minutes)

Sabrina Ali presented a computational method to explore some existing antiviral drugs as inhibitors of Severe Acute Respiratory Syndrome Coronavirus 2 (SARS-CoV-2) main protease. The molecular docking allows to filter the number of candidate molecules that are included in the post screening studies. After modelling and prediction tests, three drugs were described as the best candidates that should accelerate the identification of SARS-CoV-2 main protease inhibitors from existing antiviral drugs.

Then, Feibo Duan explained his research about an interactive holographic pyramid display system for 3D bio-images, which allows users to interact through gestures with models constructed from data obtained from microscopies. Holographic displays help researchers to understand the content of these images displaying them as a true 3D object that can be analyzed with a higher accuracy than classic 2D models.

Raúl Pérez-Moraga proposed a combination of different bioinformatics tools, which perform a comparison between SARS-CoV-2 proteins and protein structures that are known targets of Food and Drug Administration (FDA) approved drugs. To the date of this presentation, there are no drugs or therapeutic treatments approved by the FDA. Thus, drug repurposing and bioinformatics tools have emerged to address the sanitary crisis and to identify putative candidate drugs to be used as therapies for SARS-CoV-2.

In the final student flash talk of the day, Kishan KC showed InterpretablePIP, a new method to predict the probability of interaction between two proteins given their sequences. This method uses the contextual information extracted from the amino acid sequences, and a sparse gating mechanism to obtain all the positive and negative interactions between proteins.

## Round Table “Mental Health in Academia”

The ESCS2020 featured a round table focused on “Mental health in academia”. According to the World Health Organization (WHO), the concept of mental health should not be merely defined by the absence of diseases, mental disorders and/or disabilities. Instead, it must be understood as an integral state of complete physical, mental and social well-being in which the performance and abilities of an individual can cope with the normal stresses of life, thus leading to personal achievements and productive contributions to the overall community.

The aim of this panel was to engage the community in an open discussion about a concern that has been gaining increasing importance for researchers at all levels in academia: from bachelor and graduate students, to early-career researchers and professors. During this section of the event, Dr. Sofia Forslund and Dr. Patrick Aloy together with members of the ISCB-SC Executive Team Sayane Shome and Nazeefa Fatima, shared ideas, valuable insights and experiences regarding three main topics: 1) “The Burnout effect” and “Work overload” impact on the mental (and physical) health of Academics; 2) Feelings of anxiety, uncertainty and depression among graduate students; and 3) Personal coping mechanisms and institutional entities that could help to address and alleviate these conditions. The audience was also able to join the discussion through the chat, having the chance to interact with the panellists.

The “Burn out effect” describes a condition in which an individual presents diminished performance, reduced interest and health deterioration due to chronic work-related exhaustion. Inside academia this is a very common issue and despite its importance only a handful of scientific papers have been reported about this topic ( Nagy et al. 2019). For instance, according to a cross sectional study, the population of graduate students that have experienced at least one symptom of the “Burn out effect” has increased from 45% to 54% between 2011 and 2014 ( Kusurkar et al. 2020). During the panel Dr. Patrick Aloy shared some advice with the attendees about this topic. For example, he mentioned that there must be a shift in the paradigm of academic productivity from praising “hard work” to “smart work”. Furthermore, scientific productivity and impact metrics of the papers are some of the most common indicators that evaluate the performance of academics. Many times, achieving high indicator values, in combination with a lack of knowledge on project management skills, pushes academics to suffer from the “burn out effect”. In the words of Dr. Aloy: *“Some of the most productive scientists that I have known aren't the ones that work longer but the ones who administrate better their time”.* According to Dr. Aloy one important problem inside academia is that many principal investigators are trained to do quality research but they are not trained to be leaders, so the vast majority don’t have project management and soft skills that could help to guide their students effectively and pass on this knowledge. Learning from an early stage how to set goals for the research project(s), along with other activities involved in academic work, which are: Specific, Measurable, Achievable, Realistic and reached in a Timely fashion (S.M.A.R.T) could help academics to reach a high scientific productivity while not compromising their overall health ( Doran and Others 1981). Until now only a small number of graduate programs include in their programmes, courses that teach time and project management to the next generation of scientific researchers (e.g. International Max Planck Research Schools or European Molecular Biology Laboratory). Hopefully in the near future many other graduate programs will join this endeavour to prepare healthier and more productive scientific professionals.

Regarding the second topic, mental health problems such as anxiety and depression are unfortunately frequent among scientific academics. For instance, studies on this topic showed that between 43 to 46% of the graduate students from engineering, biological and physical sciences, and other related fields self-reported being depressed. Other studies showed that approximately 31.9% of the graduate students presented anxiety disorders as one of the most prevalent psychiatric disorders ( Nagy et al., 2019). In this matter Dr. Forslund shared some of her insights having coped with these issues during her research career. In the words of Dr. Forslund *“the feeling of anxiety comes from wanting to know if the steps you are taking in your professional journey will take you to the place you have envisioned as a scientist”.* The anxiety related to the academic work does not end when a paper gets published. If you want to pursue a career as a scientist (either in academia or at an enterprise), according to Dr. Forslund, you will have to find coping mechanisms to deal with the feelings of anxiety and uncertainty. This is important since other activities (e.g. obtaining funding, applying for grants, getting students and collaborations as well as other project management duties) also require you to invest time and find efficient strategies to get things done within time and economic constraints, with a good work and personal life balance. Every graduate student and early career researcher must be ready to set strategies to reduce the impact of failure and control the associated feelings of sadness and in some cases depression. Some examples from the latter may include having adequate collaborations that could help you to speed things up as well as having other back-up or side projects that will allow you to shift your attention to a different topic when a given project enters a productivity valley. Another useful skill to cope with this is to learn how to multitask and learn how to maintain an “active waiting” while expecting the results of other projects (e.g. call for applications that you are participating) along with learning and deploying self-care strategies (e.g. exercise, hobbies). Additionally and not last, it is very important to take some resting time when one starts to feel exhaustion symptoms. Taking time off to take care of oneself will always for sure help to recharge energies and come back to work with a more focused and proactive mind.

## Lessons Learned

### Perceptions of the virtual format

Overall, the experience of the attendees in the oral and poster presentations was beyond satisfactory since the online format for the presentations was set to work as pre-recorded videos and it brought many advantages. The pre-recorded format allowed presenters to rehearse their talks multiple times without the pressure of being in front of an audience and allowing them to correct specific parts of the talks if any mistake was made or something was forgotten. Pre-recorded talks also help to maintain talks within the stipulated time limits, which can be an issue when speakers become nervous or are not very experienced in giving oral presentations. This resulted to be very useful for students that are giving their first steps into presenting their work in an international setting. As a result, presentations were of really high quality in all categories. Also, this format led to a better understanding of each project since attendees had access to the recordings at all times allowing them to re-watch the content if something was not understood during the first view. Even more, attendees were allowed to make questions via chat as the presentation was being broadcasted and speakers were able to answer them immediately, extending the time for questions and discussion along the whole presentation period. In the words of Syed Muktadir Al Sium, winner in the Best Poster Presentation category: *“It was amazing to present a part of my master’s thesis and win the best poster award in a huge event like the ESCS2020. I liked the pre-recording system for poster presentation and talks. Presenters get unlimited chances to make their presentations even better. It takes the nervousness away during presentation time. Simply, this system is “Comportable” = “Comfortable” + “Portable”.*

Virtuality may not replace in-person conferences but it shows the possibility of hybrid formats even after the pandemic is over. Including a virtual aspect to in-person conferences may make them more inclusive, allowing people from all over the world to be able to present their work and participate in scientific discussions. Many of this year's attendees may not have been able to attend if the symposium was presential, due to lack of economic resources, visa restrictions or agenda clashes. Additionally, scientific presentations have traditionally favoured those people with a more extroverted profile, since they are the ones feeling more comfortable when being exposed to an audience. In contrast, more introverted people or less experienced ones, may have great work to present but the pressure of talking in public can play a negative role in their performance and hence in the way the work is communicated. Virtuality offers a novel alternative for introverts where they can prepare their presentations in the tranquillity of their homes and broadcast it during the conference. At the end of the day, the focus should be about science discussion and not how well people are adapted to talk in public.

### Importance of being prepared for unforeseen hurdles

As many projects and ventures projected to take place in 2020, our team faced the unprecedented challenges of conducting this international symposium amid the global health crisis of COVID-19. As a team, we explored new territories and considered as many possibilities as necessary to safeguard the essence and purpose of the ESCS2020. Once progressively more countries started to impose travel restrictions, our organising team decided to move the event to a virtual format. Many aspects had to be reconsidered; from selecting the best date to rescheduling deadlines and prices, planning a good promotion strategy or choosing a virtual meeting platform.

One of the biggest challenges was to obtain sponsorship. Supporting high-calibre events such as the previous congresses by the ISCB and the ISCB-SC symposia comes with many advantages for the sponsoring company such as in-place visibility of their brands. Economic activities from thousands of companies were severely disrupted due to the abrupt shutdown measures. Therefore, the possibility of having to postpone the events to safer times created some reluctance from potential sponsors to fund events. After one failed round for getting sponsors, our financial committee was able to bring Information Technology for Translational Medicine (ITTM) Solutions as a strong sponsor for ESCS2020.

Regarding the experience of attending a virtual symposium, fortunately, several technologies for the setting and transmission of online conferences were able to provide an experience closer to an in-person one. Slack was the platform selected for the communication between the event organisers. A general channel and specific ones for each subcommittee were created, allowing an efficient communication and task distribution inside and outside the subcommittees. The website content was administered by Grupal; however, some modifications in the format and appearance were difficult to implement when the event migrated from an in-person structure to an online format. After analysing alternatives, Airmeet was selected as the main platform for the event. This tool allowed us to manage different components of the online event (oral presentations, multiple rooms, discussions among participants, and a social event) in an integral and immersive manner. Additionally, Google Groups was used as the repository for the discussion of the posters, having each poster its own thread to allow the presenters to interact with the audience and solve their questions. From the view of the organising committee, the overall performance of technologies used before and during the event was satisfactory.

The main disadvantages about the organisation of an online symposium were the lack of face-to-face interactions and in-person experiences, the internet connection instabilities and the fatigue from spending many hours in front of a computer. In contrast, the main advantages were the accessibility without borders, the cost, the availability of integrative technology and the possibility to record the session. Recorded presentations can be found in a dedicated playlist for the ESCS2020 in the ISCB Youtube Channel.

### Reinforcing the promotion strategy

The outreach committee's main goal was to promote the ESCS2020 symposium to the maximum possible number of students and early career researchers working in the areas of Bioinformatics and Computational Biology ( Hassan et al. 2018). The promotion activities make sure that the targeted audience understand this event was designed for them and includes a program to address their interests. To increase the information range, we took advantage of Social Networks (Twitter, Facebook and Instagram) besides email campaigns through dedicated regional mailing lists, reaching out to the universities, known communities, and experts in the field. During the early stages of the organisation, we thoroughly planned the communications for the entire period and updated it by measuring the impact of our communications every week.

**Figure 1.**
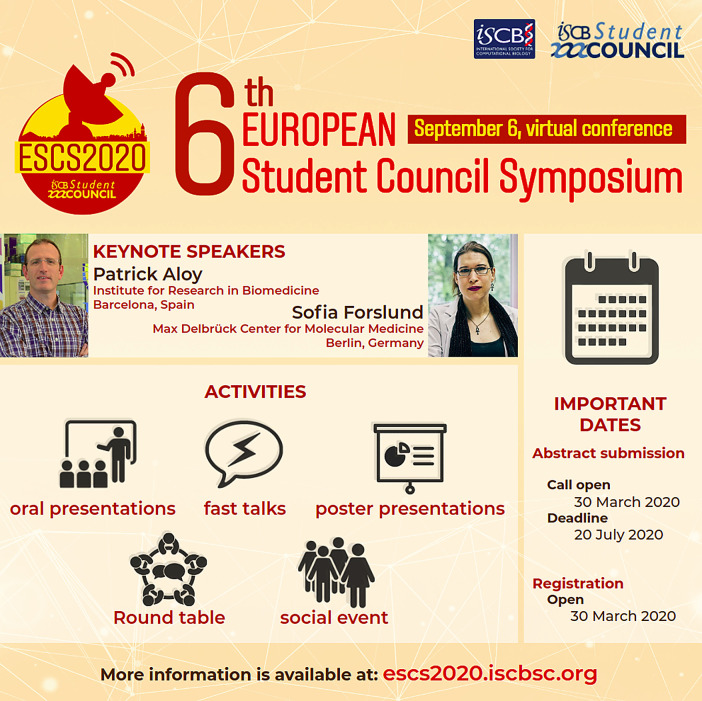
Summary flyer used in the ESCS2020 social network accounts. *Written informed consent to publish was obtained from the individuals in the flyer.*

We observed that the impact of the digital strategies grew with time and caught attendees’ attention. All the contents prepared for the outreach was shared multiple times to make it easier for targeted audiences to find the content in their timelines. We experimented with our communications by incorporating appealing visual contents (logo, banner and flyers; Figure 1) and relevant hashtags which also increased the reach and made it possible to answer the queries of the community through personalized attention. Our communication strategy also included multiple deadlines reminders and tagging the presenters accounts to show the exciting research that was going to be exposed during the Symposium. Finally, we shared media content featuring the views of the previous participants and the benefits to their career or projects by interviewing them.

During the symposium day, live-tweeting boosted the participants’ networking and discussions online. It led our communications to be released in a regular manner, resulting in a greater reach. For example, our posts through our Twitter account resulted in more than five hundred thousand tweet impressions (the number of times the tweet has been seen) and nearly four thousand profile visits. Online platforms have become a key part for disseminating and promoting scientific events. The ISCB-SC is actively working on improving its social media presence to improve the way our events are promoted in future editions.

## Award Winners

In total, six oral presentations, four flash talks and 63 poster presentations were displayed during the ESCS2020. The winners for each category were selected by voting of the attendees who submitted their vote on-line through a Google Form. The votes were registered and the winners of each category were the ones with the highest percentages of voting preference (%VP). For the category “Best Oral Presentation” Asma Yamani (King Fahd University of Petroleum and Minerals, Dhahran, Saudi Arabia) was awarded with her project titled: “ *A sequence-based approach to classify activation/inhibition relationship of protein-protein interaction (PPI) using machine learning techniques”* (27.6% VP). Two awards for the category “Best Poster Presentations” were extended to Syed Muktadir Al Sium (Bangladesh Agricultural University, Mymensingh, Bangladesh) who received the first place for his work: “ *AntiFam 6.0: Are We Close to Getting Rid of Spurious Proteins in Sequence Databases?*” (20.9% VP) and Yatindrapravanan Narasimhan (from SASTRA Deemed to be University, Thanjavur, India) who was awarded the second place through his work titled: “ *Network pharmacology of phytocompounds in Kabasurak kudineer*” (18.6% VP). Each winner received a certificate of award and an Amazon (R) Gift Card voucher as a prize according to their category. In addition, presents were also given to the keynote speakers.

## Virtual Social Event

One of the main purposes of the ESCS is to bring students and early-career researchers together and promote the networking between its members. As this was not possible to be done physically, a virtual social event was organized in the Social Lodge of Airmeet, which is a virtual room designed for the interaction among the participants of the event. The participants were able to hop between tables, meet new people and have conversations.

## Conclusions

The ISCB Student Council is the student organization of the International Society for Computational Biology with the mission to empower the next generation of computational biologists. Towards this mission, SC is supported by more than 27 ISCB Regional Student Groups that work around the globe to promote its principles and activities ( Shome et al. 2019).

From all the years the ISCB Student Council has been organising its international Symposia, due to the COVID19 pandemic, 2020 may have been by far the year that presented the biggest challenges. We faced an unprecedented situation that developed over several months with a lot of uncertainty that difficulted organisation and decision making. 2020 changed important aspects of scientific development not only related to the methods in which scientists interact and carry on their research protocols but also in the ways in which they can promote and communicate science. This was the case for ESCS2020, which was held entirely virtually for the first time, as well as all activities of the ISCB Student Council this year like the 16th SCS that was initially planned to happen in Montreal, Canada or the 4th LA-SCS in Querataro, Mexico ( Castillo-Vilcahuaman et al. 2020). Despite the initial shock received by the organising team, the experience and well predisposition from the entire ISCB-SC community allowed us, once more to take a challenging situation and turn it into a learning experience as many times before ( Mishra, Parra, and Abeel 2014).

ESCS2020 counted with an enthusiastic response of participants, keynotes and the organising team who not only ended up satisfied with the symposium outcome but also recognised several advantages in this virtual format. This leads us to anticipate the feasibility of continuing complementing the ISCB-SC Symposia with virtual activities, in order to widen the range of possibilities to communicate science as an option to deal with future adversities and to incorporate attendees coming from all over the globe ( “Are Hybrid Events the Future of Academic Conferences?” 2020) ( “Hybrid Meeting” n.d.).

As the organising team of the ESCS2020, we know that like never before computational scientists, in particular computational biologists, have been fundamental for the development of new policies in which healthcare, respect for the environment, and sustainable production are the fundamental principles. A cohesive global community of computational biologists is imperative to accomplish these goals and we will continue working strongly to achieve them.

If you are interested in collaborating with the ISCB-SC, do not hesitate to contact the RSG of your country or work towards establishing a new one focused on the particular needs of your local computational biology and bioinformatics communities.

## Data Availability

No data is associated with this article.
